# Increased Transmission of *Mycobacterium tuberculosis* Beijing Genotype Strains Associated with Resistance to Streptomycin: A Population-Based Study

**DOI:** 10.1371/journal.pone.0042323

**Published:** 2012-08-13

**Authors:** Tran N. Buu, Dick van Soolingen, Mai N. T. Huyen, Nguyen T. N. Lan, Hoang T. Quy, Edine W. Tiemersma, Kristin Kremer, Martien W. Borgdorff, Frank G. J. Cobelens

**Affiliations:** 1 Tuberculosis Control Department, Pham Ngoc Thach Hospital, Ho Chi Minh City, Vietnam; 2 Laboratory for Infectious Diseases and Screening, National Institute of Public Health and The Environment, Bilthoven, The Netherlands; 3 Department of Medical Microbiology, University Medical Center Saint Radboud, Nijmegen, The Netherlands; 4 Microbiology Department, Pham Ngoc Thach Hospital, Ho Chi Minh City, Vietnam; 5 Medical Department, Pham Ngoc Thach Hospital, Ho Chi Minh City, Vietnam; 6 Asia Department, KNCV Tuberculosis Foundation, The Hague, The Netherlands; 7 Amsterdam Institute of Global Health and Development, Amsterdam, The Netherlands; 8 Division of Communicable Diseases, Health Security & Environment,World Health Organization Regional Office for Europe, Copenhagen, Denmark; 9 Infectious Diseases Cluster, Amsterdam Municipal Health Service, Amsterdam, The Netherlands; 10 Department of Global Health, Academic Medical Center, Amsterdam, The Netherlands; St. Petersburg Pasteur Institute, Russian Federation

## Abstract

**Background:**

Studies have shown that the *Mycobacterium tuberculosis* Beijing genotype is an emerging pathogen that is frequently associated with drug resistance. This suggests that drug resistant Beijing strains have a relatively high transmission fitness compared to other drug-resistant strains.

**Methods and Findings:**

We studied the relative transmission fitness of the Beijing genotype in relation to anti-tuberculosis drug resistance in a population-based study of smear-positive tuberculosis patients prospectively recruited and studied over a 4-year period in rural Vietnam. Transmission fitness was analyzed by clustering of cases on basis of three DNA typing methods. Of 2531 included patients, 2207 (87%) were eligible for analysis of whom 936 (42%) were in a DNA fingerprint cluster. The clustering rate varied by genotype with 292/786 (37%) for the Beijing genotype, 527/802 (67%) for the East-African Indian (EAI) genotype, and 117/619 (19%) for other genotypes. Clustering was associated with the EAI compared to the Beijing genotype (adjusted odds ratio (OR^adj^) 3.4: 95% CI 2.8–4.4). Patients infected with streptomycin-resistant strains were less frequently clustered than patients infected with streptomycin-susceptible strains when these were of the EAI genotype (OR^adj^ 0.6, 95% CI 0.4–0.9), while this pattern was reversed for strains of the Beijing genotype (OR^adj^ 1.3, 95% CI 1.0–1.8, p for difference 0.002). The strong association between Beijing and MDR-TB (OR^adj^ 7.2; 95% CI 4.2–12.3) existed only if streptomycin resistance was present.

**Conclusions:**

Beijing genotype strains showed less overall transmissibility than EAI strains, but when comparisons were made within genotypes, Beijing strains showed increased transmission fitness when streptomycin-resistant, while the reverse was observed for EAI strains. The association between MDR-TB and Beijing genotype in this population was strongly dependent on resistance to streptomycin. Streptomycin resistance may provide Beijing strains with a fitness advantage over other genotypes and predispose to multidrug resistance in patients infected with Beijing strains.

## Introduction

Twenty years after the introduction of WHO's DOTS strategy, tuberculosis (TB) remains a common and often deadly infectious disease. In 2010, there were an estimated 9.4 million incident cases of TB with more than 1.8 million deaths around the world [1]. Resistance of *Mycobacterium tuberculosis* to anti-tuberculosis drugs is one of the major challenges to TB control, particularly multi-drug resistance (MDR), defined as resistance to at least isoniazid and rifampicin, the two most powerful first-line anti-TB drugs [2]. MDR-TB is associated with high rates of failure and death when treated with the standard first-line treatment regimens, as is common in most high-burden countries [3]. In 2008, the WHO estimated 3.6% of all incident TB cases globally to have MDR-TB, with the proportion of MDR-TB ranging from 0% to 28% among new, and from 0% to 61.6% among previously treated TB patients [3].

Although resistance to TB drugs is considered a man-made amplification of a natural phenomenon due to inadequate treatment regimens and incomplete treatment adherence [4], recent studies have suggested an additional role of the causative bacteria, in particular for *M. tuberculosis* Beijing genotype strains. First described in 1995, the Beijing genotype probably originates from East Asia but has been encountered in many countries worldwide, notably the former Soviet Union where major problems with anti-TB drug resistance exist [5], [6]. This genotype has also been associated with drug resistance in other areas [5], [7], [8]. In studies from Europe, South Africa, Taiwan and Malawi, Beijing strains were predominant among young age groups, suggesting recent spread and emergence [8], [9], [10], [11], [12], [13], while data from the Gambia indicated that Beijing strains may have shorter incubation periods than other genotypes [14]. Molecular analyses suggest that this genotype has higher frequencies of particular drug resistance-conferring mutations, possibly due to alterations in genes that prevent mutation in genes encoding for drug resistance [15], [16], [17], [18]. This may imply that Beijing strains have an increased transmission fitness compared to other genotypes of *M. tuberculosis* that predominate in these settings. This increased fitness could play a role for all forms of resistance in general or especially for strains that display (multi)drug-resistance. Such increased fitness would be reflected in e.g. increased transmissibility and thus increased rates of DNA fingerprint clustering [8], [9], [10], [11], [12], [13], [14], [15] though several studies assessed fingerprint clustering of drug-resistant strains or strains of specific genotypes, few have looked at both in a population-based design.

Vietnam is one of 22 high burden countries in the world [1]. The prevalence of MDR-TB among new TB patients is around 2.4%, but considerably higher in previously treated cases [1], [3]. Previous studies have shown that the prevalence of *M. tuberculosis* Beijing genotype in Vietnam was 53% in urban and 35% in rural areas [19], [20]. This genotype was found to be more frequent in young patients, strongly associated with MDR-TB, and possibly associated with relapse [21], [22]. We therefore hypothesized that the Beijing genotype has increased transmission potential when (multi-)drug-resistant in comparison to other strains circulating in Vietnam.

In order to test this hypothesis, we assessed the relative transmission fitness of Beijing strains with and without drug resistance by analysing DNA fingerprint cluster data of a population-based study in a rural area of Vietnam.

## Methods

### Study population and design

The study area consisted of three adjacent rural districts in Tien Giang Province, situated in the Mekong River Delta in southern Vietnam with a total population of 895,863, and a notification rate of smear-positive TB of 100/100,000 (2003). Each has a district TB unit (DTU) that performs sputum smear examinations and treats ambulatory smear-positive patients according to the DOTS strategy. Patients with severe smear-positive disease, as well as suspects of smear-negative or extra-pulmonary TB, are referred for diagnosis and treatment initiation to the provincial TB hospital. There are no other laboratories that perform smear examination, and private physicians do not treat TB. Population movement is limited; the non-resident population was estimated at around 1%. In 2001 the MDR-TB prevalence in the south of Vietnam was 1.8% among new and 23% among previously treated patients [23]. HIV testing of TB patients is done on clinical suspicion only.

Diagnosis of smear-positive TB was by microscopic examination of at least two Ziehl-Neelsen stained sputum smears [24]. Eligible for inclusion were all patients aged 15 years or more who were resident in the study area and registered for treatment of smear-positive pulmonary TB at the participating DTU's or at the provincial TB hospital between 1 January 2003 and 31 December 2006. Patients in one of the districts were only eligible from 1 October 2003 onwards. Eligible patients were included upon provision of written informed consent. Excluded were patients who had been under treatment of the current TB episode for more than two weeks before inclusion. Scientific and ethical clearance was obtained from the Ethical Health Committee of the Ho Chi Minh City Council.

Enrolled patients were interviewed using a standard questionnaire including items on socio-demographic information, details of the patient's households, clinical data, details of any previous TB treatment, and history of possible infectious contact. Each patient submitted two sputum specimens for mycobacterial culture that were kept refrigerated and were transported to the Mycobacterial Reference Laboratory in Ho Chi Minh City within 72 hours.

### Laboratory methods

At the Reference Laboratory, sputum specimens were decontaminated and liquefied with 1% N-Acetyl-L-Cysteine (NALC) - 2% NaOH, inoculated on modified Ogawa medium and incubated at 37°C [25]. Cultures with no growth after eight weeks were reported as negative. *M. tuberculosis* was identified by the niacin and nitrate tests. Drug susceptibility testing (DST) was done by the proportion method following WHO/IUATLD guidelines. Criteria for drug resistance were ≥1% colony growth at 28 or 40 days compared to the drug-free control medium at the following drug concentrations: isoniazid (H) 0.2 µg/ml, rifampin (R) 40 µg/ml, streptomycin (S) 4 µg/ml and ethambutol (E) 2 µg/ml [25]. DNA was extracted from *M. tuberculosis* cultures and genotyped by spoligotyping [26], IS*6110*-based restriction fragment length polymorphism (RFLP) typing [27] and 15-loci variable numbers of tandem repeats (VNTR) typing [28], [29]. IS*6110* RFLP and spoligotype patterns were analyzed by using Bionumerics software (Applied Maths, Sint-Martens-Latum, Belgium) as described previously [30]. Similarity between the DNA fingerprint patterns was calculated by using the Dice coefficient with 1% position tolerance and optimization, and UPGMA for clustering.

### Definitions

The Beijing genotype was defined by spoligotyping as any isolate without hybridization to spacers 1–34 and the presence of ≥3 of the spacers 35–43 [6]. Other genotypes were defined as described by Brudey et al [31], including the Vietnam genotype that belongs to the East-African Indian (EAI) genotype family of *M. tuberculosis* (designated EAI4-VNM) and is the most frequent genotype in this study site [20].

A cluster was defined as ≥2 *M. tuberculosis* isolates sharing identical or highly similar DNA fingerprints on basis of three independent genetic markers [32]. We used criteria for clustering based on the combination of results of IS*6110* RFLP typing, spoligotyping and VNTR typing. Only 100% identity was used for clustering of IS*6110* RFLP and spoligotype patterns. VNTR patterns were considered similar if ≤1 VNTR locus displayed more than one allele, and different when ≥2 loci displayed more than one allele.

We defined *M. tuberculosis* infections as multiple >1 strain was detected in a single patient isolate, based on discordant spoligo and RFLP patterns and/or multiple VNTR loci with ≥1 allele [33]. Previous treatment was defined a history of anti-tuberculosis treatment for ≥1 month [24]. A recurrent TB case was defined as one that had been previously treated for TB with ‘treatment completed’ or ‘default’ as the outcome. Re-infection was a case of recurrent TB in which the isolated strain differed from that isolated in the previous episode, while for relapse the strains was the same, based on the definition for clustering mentioned above.

### Data management and analysis

Data were entered in Epi-Info (version 6.04; Centers for Disease Control and Prevention, Atlanta GA). Double entry was done on a 20% random sample (50% for 2003) of all records. Discrepancies were observed in <1% of all records, and in <0.05% of all fields. Analyses were performed in Stata (version 8; Stata Corp., College Station TX). Patients with negative cultures or cultures that grew non-tuberculous mycobacteria were excluded from the analyses, as were patients with multiple infections or relapses.

In cases that presented with a double allele in one VNTR locus, we based the cluster definition on one of these, and did a secondary analysis in which we based the cluster definition on the second allele at that locus. In the analyses, we compared the proportions of patients who belonged to a cluster (i.e. shared a fingerprint with ≥1 other patient in the database) to those of patients who did not belong to a cluster (unique cases).

Of 210 strains belonging to spoligotype family EAI4 that were initially typed by RFLP, 189 (86%) had <5 IS*6110* copies [31], which limited the use of RFLP typing with regard to defining transmission clusters within this group. Therefore we stopped RFLP typing of the isolated strains except for those with a Beijing spoligotype halfway the enrolment period. In the cluster analyses, we made the assumption that all EAI strains for which no RFLP data were available had <5 IS*6110* copies and thus belonged to the same RFLP type, and that all strains with genotypes other than EAI or Beijing (“other”) for which no RFLP data were available had unique RFLP types. We verified this assumption by estimating for each of the three major genotype groups (EAI, Beijing and other) the numbers of strains not typed that were falsely classified as being similar or different assuming that the isolates for which we had RFLP data were a random selection of all isolates with regard to the number (>5 or ≥5) of IS*6110* copies.

For significance testing of comparisons of categorical variables, the chi-square test or the two-sided Fisher's exact test was used as appropriate. Multivariate analyses were done by logistic regression modeling. P-values for contribution to multivariate models, including interaction, were based on the likelihood ratio chi-squared test; p-values for contribution to models of individual strata of variables were based on the Wald test; p-values for the interaction of drug resistance on association between genotype (Beijing vs. EAI) and MDR-TB were based on Mantel-Haenszel stratification. All tests were done at the 5% significance level.

## Results

### Study population

**Figure 1:**
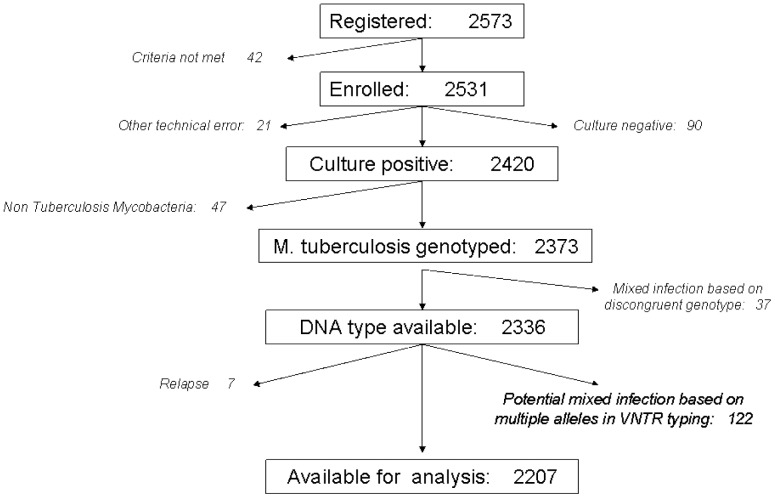
Study flow chart of patient inclusion.

Over the 4 year study period, 2573 smear-positive pulmonary TB patients were registered for treatment, of whom 2531 met the inclusion criteria. Twenty-one were excluded for technical errors, 90 for negative cultures and 47 for isolation of non-tuberculous mycobacteria. After DNA typing of the remaining 2373 (92.2%) isolates we excluded seven relapse cases and 159 mixed infections, leaving 2207 patients for the analyses (87.2% of those enrolled; Figure 1). Excluded patients were significantly older, more often female and more often previously treated than included patients (Table 1). RFLP information was lacking for 534 isolates including four Beijing, 335 EAI, and 195 other genotype strains. The estimated proportions falsely classified as similar were 0.3% for Beijing strains and 3.0% for EAI strains, whereas the proportion of other genotype strains falsely classified as different was 16.0%.

**Table 1:** Characteristics of the patients included or excluded from the study among the 2573 enrolled patients.

	Included patients		Excluded patients		p value
	N	% distribution	N	% distribution	
Total	2207		366		
Year of inclusion					
2003	427	19.3	82	22.4	0.167
2004	638	28.9	103	28.1	0.764
2005	573	26.0	91	24.9	0.643
2006	569	25.8	90	24.6	0.629
District of residence					
Cai Be	662	30.0	103	28.1	0.472
Cai Lay	910	41.2	152	41.5	0.902
Chau Thanh	635	28.8	111	30.3	0.555
Age (years)					
<25	182	8.2	19	5.2	0.046
25–34	286	13.0	32	8.7	0.022
35–44	455	20.6	65	17.8	0.208
45–54	454	20.6	60	16.4	0.615
55–64	246	11.1	47	12.8	0.331
65+	584	26.5	143	39.1	<0.001
Sex					
Male	1654	74.9	249	68.0	0.006
Female	553	25.1	117	32.0	
History of TB treatment					
New	1987	90.0	316	86.3	0.035
Previously treated	220	10.0	50	13.7	

### Clustering by genotype

Of the 2207 cases, EAI strains accounted for 36.3% (802 patients), Beijing strains for 35.6% (786) and other genotypes for 28.1% (619). The proportion Beijing strains increased slightly from 33.8% in the first year to 38% in the fourth year, whereas the proportion EAI strains remained the same (33.8% to 33.1%) and the proportion other strains decreased from 32.2% to 28.6%; these differenced were not significant (p = 0.323). MDR was strongly associated with the Beijing genotype: after multivariable adjustment for year of inclusion, treatment history, residence, sex and age the odds of MDR was 7.2 times higher among Beijing (68/786 cases, 8.7%) than among EAI or other genotypes (18/1421 cases, 1.3%; 95% CI 4.2–12.3, p<0.001).

We identified 936 patients (proportion; 95% CI) as being in one of 245 clusters (42.4%; 40.4–44.5), with cluster sizes ranging from two to 83 patients. Among patients infected with EAI genotypes, 527 were clustered (65.7%; 62.4–68.9) in 96 different clusters, accounting for 39.2% of the total number of clusters. The proportions in clusters were 292/786 (37.2%; 33.8–40.6) among patients infected with Beijing strains and 117/619 (18.9%; 16.0–22.1) among patients infected with other genotypes (Table 2). The Beijing genotype accounted for 42.9% of the total number of clusters (105/245) and predominantly occurred in cluster sizes of two (69/140) while EAI predominantly occurred in cluster sizes of three or more. Cluster of 10 or more cases were observed for EAI strains (including one of 10, 16, 22 and 35 cases, respectively, and two of 15 cases each), but not for Beijing and other genotype strains.

**Table 2:** Genotype and cluster distribution based on combined clustering of IS*6110* RFLP, spoligotyping and VNTR typing, among smear-positive pulmonary TB patients in rural Vietnam, 2003–2006.

Genotypes/sub-genotypes as defined by spoligotyping^1^		Isolated strains		In cluster	
		n	%	n	% (95% CI)
BEIJING		786	35.6%	292	37.2 (3.8–40.6)
	Beijing	782	35.4%	290	37.1%
	Beijing-like	4	0.20%	2	50.0%
EAI		802	36.3%	527	65.7 (62.4–68.9)
	EAI4 VNM	389	17.6%	301	77.4%
	EAI5	300	13.6%	203	67.7%
	EAI2 MANILLA	71	3.2%	13	18.3%
	EAI1 SOM	30	1.4%	10	20.0%
	EAI2 NTB	11	0.5%	0	0.0%
	EAI (undefined)	1	0.0%	0	0.0%
OTHERS		619	28.1%	117	18.9% (16.0–22.1)
	NA	337	15.3%	34	10.1%
	U	75	3.4%	11	14.7%
	U(LIKELY H)	2	0.1%	0	0.0%
	ZERO	67	3.0%	23	34.3%
	T1	54	2.5%	26	48.2%
	T2	17	0.8%	2	11.8%
	T2T3	3	0.1%	0	0.0%
	T3	4	0.2%	0	0.0%
	T5	2	0.1%	0	0.0%
	H3	38	1.7%	14	36.8%
	H1	3	0.1%	0	0.0%
	S	6	0.3%	5	83.3%
	LAM9	2	0.1%	0	0.0%
	MANU1	1	0.0%	0	0.0%
	MANU2	1	0.0%	0	0.0%
	CAS	2	0.1%	0	0.0%
	CAS1DELHI	2	0.1%	0	0.0%
	CAS1KILI	2	0.1%	0	0.0%
	X2	1	0.0%	0	0.0%
TOTAL		2,207	100%	936	42.4% (40.4–44.5)

1 Genotype or subgenotype classification was based on spoligotyping classification by Brudey *et al.* (SPOL4 database).

### Risk factors for clustering

In univariate analyses, compared to Beijing genotype infections, clustering was more frequent with EAI genotype infections (odds ratio (OR) 3.2; 95% CI 2.6–5.0) but less frequent with other genotypes (0.4; 0.3–0.5). Clustering was also less frequent, albeit non-significant, among streptomycin-resistant strains (0.9; 0.7–1.0), ethambutol-resistant strains (0.8; 0.3–1.3) and MDR strains (0.8; 0.5–1.2) (Table 3). Clustering was not associated with history of TB treatment, year of inclusion, socio-demographic characteristics (age, sex, residence, level of education, occupation, marital status, family size) or the amount of time spent in places with potential TB exposure such as markets, restaurants, offices, hospitals, factories, schools, public transportation and prisons (data not shown).

**Table 3:** Univariable and multivariable associations of fingerprint clustering with socio-demography, history of tuberculosis treatment and drug resistance among 2207 smear-positive pulmonary tuberculosis patients in Vietnam, 2003–2006.

	Total	Patients in clustern (%)	Crude OR	p value	AdjustedOR^1^ (95% CI)	p value^2^
All patients	2207	936 (42.4%)				
Genotype				<0.001		<0.001
Beijing	786	292 (37.2%)	1		1	
EAI	802	527 (66.7%)	3.2		3.4 (2.8–4.4)	
Others	619	117 (18.9%)	0.4		0.4 (0.3–0.5)	
Resistance to streptomycin				0.094		0.470
No	1617	703 (43.5%)	1		1	
Yes	590	233 (39.5%)	0.9		1.1 (0.9–1.4)	
Resistance to ethambutol				0.484		0.650
No	2169	922 (42.5%)	1		1	
Yes	38	14 (36.8%)	0.8		1.2 (0.5–2.7)	
Multidrug resistance				0.223		0.611
No	2121	905 (42.7%)	1		1	
Yes	86	31 (36.0%)	0.8		0.9 (0.5–1.5)	
TB treatment history				0.365		0.651
New	1987	849 (42.7%)	1		1	
Previously treated	220	87 (39.5%)	0.9		1.1 (0.8–1.5)	
Year of inclusion				0.143		0.071
2003	427	181 (42.4%)	1		1	
2004	638	264 (41.4%)	1.0		0.9 (0.7–1.2)	
2005	573	265 (46.2%)	1.2		1.1 (0.9–1.5)	
2006	569	226 (39.7%)	0.8		0.8 (0.6–1.1)	
Age (years)				0.088		0.105
<25	182	72 (39.6%)	1		1	
25–34	286	116 (40.6%)	1.0		1.0 (0.6–1.5)	
35–44	455	213 (46.8%)	1.3		1.2 (0.8–1.7)	
45–54	454	207 (45.6%)	1.3		1.1 (0.7–1.5)	
55–64	246	94 (38.2%)	0.9		0.8 (0.5–1.2)	
65+	584	234 (40.1%)	1.0		0.9 (0.6–1.2)	
Sex				0.344		1.000
Male	1654	771 (46.6%)	1		1	
Female	553	225 (40.7%)	0.8		1.0 (0.8–1.2)	
Commune of residence				0.091		0.267
On waterway only	837	378 (45.2%)	1		1	
On provincial road	1133	456 (40.2%)	0.8		0.9 (0.7–1.0)	
On national road	237	102 (43.0%)	0.9		1.0 (0.7–1.4)	

ORs: Odd ratios CI: confidence interval.

1 Odd ratios adjusted by logistic regression for year of inclusion and all variables in the model.

2 P values based on likelihood ratio test for excluding variable from logistic regression model.

After multivariable adjustment for year of inclusion, treatment history, drug resistance, residence, sex and age, the risk of clustering remained significantly associated (adjusted OR (OR^adj^); 95% CI) with genotype (EAI vs. Beijing: 3.4; 2.8–4.4; other genotypes vs. Beijing: 0.4; 0.3–0.5). Associations with drug resistance disappeared or became non-significant. We found no significant interactions between genotype and drug resistance including MDR.

### Effect of drug resistance on clustering by genotype

In order to compare the effects of drug resistance on the extent of clustering within genotypes, we stratified the analysis by the two predominant genotypes, i.e. EAI and Beijing, again taking the proportion of clustered cases as the outcome, and adjusting the associations for differences in year of inclusion, age, sex, residence and previous TB treatment (Table 4). Among patients with Beijing genotype infections, clustering was significantly more frequent if the infecting strain was resistant than if the infecting strain was susceptible to streptomycin (OR^adj^ 1.3; 95% CI 1.0–1.8, p = 0.041), to isoniazid (1.5; 1.1–2.1, p = 0.022) or to streptomycin and isoniazid combined (1.5; 1.1–2.1, p = 0.023). Conversely, among patients with EAI genotype infections clustering was less frequent if the infection strain was resistant than if the infecting strain was susceptible to streptomycin (0.6; 0.4–0.9, p = 0.016) or to streptomycin-isoniazid (0.6; 0.4–1.1, p = 0.371, while there was no difference in proportion clustering between EIA strains that were resistant and EIA strains that were susceptible to isoniazid only (0.9; 0.6–1.5, p = 0.912). The differences between Beijing and EIA strains were statistically significant (p values for interaction between resistance and genotype) for streptomycin resistance (0.002) and for combined streptomycin-isoniazid resistance (0.021). No clearly diverging patterns of the extent of clustering between EAI and Beijing strains were observed for resistance to rifampicin or ethambutol, or for MDR (Table 4).

**Table 4:** Association between fingerprint clustering and *M. tuberculosis* genotype (Beijing vs. East-African-Indian) by drug resistance patterns among smear-positive pulmonary tuberculosis in rural Vietnam, 2003–2006.

Drug resistance	East-African-Indian genotypes			Beijing genotypes			p value for interaction 2
	Total	In cluster n (%)	Adjusted OR 1(95% CI)	Total	In cluster n (%)	Adjusted OR^1^ (95% CI)	
Streptomycin							
Susceptible	711	478 (67.2%)	1	398	134 (33.7%)	1	0.002
Resistant	91	49 (53.9%)	0.6 (0.4–0.9)	388	158 (40.7%)	1.3 (1.0–1.8)	
Isoniazid							
Susceptible	697	458 (65.7%)	1	555	192 (34.6%)	1	0.140
Resistant	105	69 (65.7%)	0.9 (0.6–1.5)	231	100 (43.3%)	1.5 (1.1–2.1)	
Rifampicin							
Susceptible	792	520 (66.7%)	1	706	260 (36.8%)	1	0.925
Resistant	10	7 (70.0%)	1.0 (0.3–4.2)	80	32 (40.0%)	1.2 (0.7–1.9)	
Ethambutol							
Susceptible	799	525 (65.7%)	1	760	281 (37.0%)	1	0.822
Resistant	3	2 (66.7%)	1.0 (0.1–12.0)	26	11 (42.3%)	1.3 (0.6–2.9)	
Streptomycin and isoniazid							
Susceptible	750	479 (66.3%)	1	575	200 (34.8%)	1	0.021
Resistant	52	30 (57.7%)	0.6 (0.4–1.1)	211	92 (43.6%)	1.5 (1.1–2.1)	
Rifampicin and isoniazid							
Susceptible	795	522 (65.7%)	1	718	267 (37.2%)	1	0.875
Resistant	7	5 (71.4%)	1.2 (0.2–6.5)	68	25 (36.8%)	1.0 (0.6–1.7)	

ORs: Odds ratios, with 95% confident interval CI: Confidence interval.

1 Adjusted by logistic regression modeling for year of inclusion, age, sex, commune of residence and history of tuberculosis treatment.

2 P values based on Wald test for comparison of stratum to reference category. Denotes the level of significance for the difference between East-African-Indian and Beijing genotypes in the association of clustering and drug resistance.

### Effect of streptomycin resistance on MDR

The apparently increased reproductive fitness of streptomycin-resistant Beijing strains prompted us to further investigate the role of streptomycin resistance in the association between MDR-TB and genotype. We therefore stratified the adjusted relative risk of MDR-TB among Beijing vs. non-Beijing genotypes by streptomycin and combined streptomycin-isoniazid resistance status. Multivariate adjustment was again for age, sex, year of inclusion and residence. We found that streptomycin resistance as well as combined streptomycin-isoniazid resistance strongly confounded the association between MDR-TB and genotype among both all and new patients. While among all patients those with Beijing strains had a 7.2-fold increased risk of MDR-TB compared to non-Beijing strains, this risk was only 2.4 and 2.6 times increased after adjustment for differences in streptomycin resistance and in combined streptomycin-isoniazid resistance, respectively (Table 5). Similarly, among new patients adjustment for resistance to streptomycin and streptomycin-isoniazid resistance decreased the relative risk of MDR-TB among Beijing infected patients from 8.8 to 2.9 and 3.0, respectively. In each comparison there was significant interaction between streptomycin or streptomycin-isoniazid resistance status and genotype. Further stratification showed that the association between Beijing genotype and MDR-TB completely depended on resistance to streptomycin: none of the streptomycin-susceptible Beijing strains was MDR (Table 6).

**Table 5:** The role of streptomycin resistance and combined streptomycin-isoniazid resistance on the association between *M. tuberculosis* genotype (Beijing vs. non-Beijing) and multi-drug resistance among smear-positive pulmonary TB patients in rural Vietnam, 2003–2006.

	Total	MDR-TBn (%)	OR(95% CI)^1^	OR adjusted for resistance to streptomycin(95% CI)^2^	OR adjusted for combined resistance to streptomycin and isoniazid(95% CI)^3^
All smear positive pulmonary TB					
Non Beijing	1421	18 (1.3%)	1	1	1
Beijing	786	68 (8.7%)	7.2 (4.2–12.3)	2.4 (1.4–4.2)	2.6 (1.4–4.6)
New smear positive pulmonary TB					
Non Beijing	1324	10 (0.8%)	1	1	1
Beijing	663	42 (6.3%)	8.8 (4.4–17.9)	2.9 (1.4–6.1)	3.0 (1.4–6.5)

ORs: Odds ratios, CI: Confidence interval.

MDR-TB: multi-drug resistance tuberculose.

1 Adjusted for age, sex, year of inclusion, commune of residence by logistic multivariate regression model.

2 Adjusted for Age, sex, year of inclusion, commune of residence and streptomycin resistance by logistic multivariate regression model.

3 Adjusted for Age, sex, year of inclusion, commune of residence and combined resistance to streptomycin and isoniazid by logistic multivariate regression model.

**Table 6:** Association between multi-drug resistance and *M. tuberculosis* genotype (Beijing vs. non Beijing) stratified by resistance to streptomycin and combined resistance to streptomycin and isoniazid, among smear-positive pulmonary TB patients in rural Vietnam, 2003–2006.

	Total	MDR-TB	OR (95% CI)	p value^1^
All smear positive pulmonary TB				
Non BJ and streptomycin susceptible	1219	4 (0.3%)	1	
BJ and streptomycin susceptible	398	0 (0.0%)	0.0 (0.0–2.9)	0.063
Non BJ and streptomycin resistant	202	14 (6.9%)	1	
BJ and streptomycin resistant	388	68 (17.5%)	2.9 (1.5–5.6)	
Non BJ and SH susceptible	1313	4 (0.3%)	1	
BJ and SH susceptible	575	0 (0.0%)	0.0 (0.0–2.2)	0.026
Non BJ and SH resistant	108	14 (13.0%)	1	
BJ and SH resistant	211	68 (32.2%)	2.9 (1.7–6.5)	
New smear positive pulmonary TB				
Non BJ and streptomycin susceptible	1153	3 (0.3%)	1	
BJ and streptomycin susceptible	358	0 (0.0%)	0.0 (0.0–4.13)	0.078
Non BJ and streptomycin resistant	171	7 (4.1%)	1	
BJ and streptomycin resistant	305	42 (13.8%)	3.7 (1.6–10.10)	
Non BJ and SH susceptible	1240	3 (0.2%)	1	
BJ and SH susceptible	509	0 (0.0%)	0.0 (0.0–3.1)	0.038
Non BJ and SH resistant	84	7 ( 8.3%)	1	
BJ and SH resistant	154	42 (27.3%)	4.1 (1.7–11.4)	

BJ: Beijing genotype.

S: streptomycin H: isoniazid.

MDR-TB: multi-drug resistant tuberculosis.

ORs: Odds ratios, CI: Confidence interval.

1 P values for interaction from Mantel-Haenszel'stratification. Denotes statistical significance of the difference between streptomycin(-isoniazid) susceptible and resistant status of the association between multidrug resistance and genotype.

### Additional analyses

When we defined clusters by VNTR and spoligotyping only, the proportion of patients in clusters increased to 80.4% (1774/2207). This increase was substantial for Beijing and other genotypes from 37.2% to 98.6% and from 18.9% to 52.7%, respectively, but less so for EAI genotypes (from 66.7% to 83.9%). Consequently, the rate of clustering became lower for EAI than for Beijing genotype strains, both before (OR 0.07) and after multivariable adjustment as described above (OR^adj^ 0.08; 95% CI 0.04–1.16, p<0.001). Moreover, multidrug resistance appeared as a strong risk factor for clustering (OR^adj^ 6.2; 95% CI 1.1–34.8, p = 0.017). With this limited cluster definition, the divergence between Beijing and EAI genotypes with respect to the association between clustering and streptomycin resistance became more pronounced: among patients infected with Beijing genotype strains streptomycin resistance increased the proportion clustering by 2.9-fold (OR^adj^; 95% CI 0.7–11.4), while among patients infected with EAI genotype strains streptomycin resistance decreased the this proportion by 0.7-fold (0.4–1.2). This difference between the genotypes remained significant (p = 0.050). Similarly, more pronounced differences in proportions clustering between EAI and Beijing genotype strains were observed for resistance to isoniazid and to ethambutol, as well as for combined resistance to streptomycin and isoniazid; these did however not reach statistical significance (data not shown).

## Discussion

In 42% of all patients the isolated strain had a DNA fingerprint that clustered with that of at least one other patient. Beijing strains in general, i.e. not taking into account specific patterns of drug resistance, displayed less clustering than EAI strains. This suggests that Beijing strains are overall less transmissible than EAI strains. However, because many of the EAI strains had few copies of IS*6110*, the three-methods typing system we used may have had reduced ability for discriminating EAI strains, resulting in an overestimation of the extent of clustering for this genotype. That such overestimation may have occurred was suggested by the secondary analysis of fingerprint clusters based on VNTR and spoligotyping only, which showed significantly more clustering for Beijing than for EAI strains. One the other hand, this two-method typing system may have overestimated the extent of clustering for Beijing strains. Hence, our data cannot be considered conclusive with regard to the overall transmission fitness of Beijing versus EAI and other genotype trains.

Since the discriminatory power of the typing system is expected to be similar within each genotype, we also looked at the proportion clustering within the strata of the Beijing and EAI genotypes separately. Strains that were resistant to streptomycin, isoniazid or streptomycin and isoniazid combined showed higher tendency for clustering than susceptible strains if they were of the Beijing genotype, but not if they were EAI genotype. EIA strains that were resistant to streptomycin were even significantly less often clustered than EIA strains that were susceptible to streptomycin. This suggests that while for EAI strains resistance to streptomycin or streptomycin-isoniazid results in reduced transmissibility, Beijing strains with these resistances have in fact increased transmissibility, pointing to fitness differences between resistant strains of these genotypes. The apparent key role for streptomycin resistance is further supported by our finding that in this setting the association between the Beijing genotype and MDR-TB to large extent depended on the presence of streptomycin resistance.

The overall proportion of clustering in our study was similar to that found in Spain (42%) and Germany (49%), but lower than clustering proportions in Russia (60%), Taiwan (67%) and South Africa (72%) [9], [34], [35], [36], [37]. This may reflect differences in transmission: our study was performed in a rural area while the others were done in urban areas (Russia, South Africa) or in hospitals (Taiwan, Spain) where transmission tends to be higher. More likely, however, it reflects differences in typing methods. Other studies used monotyping methods or combined two methods, while we based our definition of fingerprint clusters on a combination of three; IS*6110* RFLP, spoligotyping and VNTR typing. Our approach thereby maximized the likelihood that a cluster represents epidemiologically related strains, but inherently minimized the number of transmission clusters identified [9], [38], [39]. Furthermore, when we used only spoligotyping and VNTR typing results the proportion of clustered Beijing strains increased, while this did not happen for EAI strains. Thus, VNTR clusters of Beijing strains were split up by RFLP typing to considerable extent, suggesting that VNTR typing has reduced discriminatory power for Beijing strains and therefore analyses based on VNTR and spoligotyping only falsely increase the proportion clustering among Beijing strains. Comparing the results of the two- versus the three-method typing system it thus seems that our study is inconclusive with regard to the overall relative reproductive fitness of Beijing versus EAI strains. On the other hand, the pattern of differences between Beijing and EAI genotypes in the extent of clustering of resistant versus susceptible strains remained the same and even became more pronounced when we defined clusters based on VNTR and spoligotyping only, which indicates that this finding was robust to the typing system used.

Our data suggest that streptomycin-resistant strains of the Beijing genotype have no reduced, or even increased, reproductive fitness. This finding is somewhat similar to that of studies from The Netherlands and San Francisco that showed no reduced clustering of isoniazid-resistant strains if they harbored the S315T mutation in the katG gene, although in those studies no genotype association was proven [40], [41]. It prompted us to reassess our earlier finding from this study site (on a then smaller dataset) of the strong association between MDR and the Beijing genotype for an underlying association with streptomycin-resistance [20], which we indeed confirmed. We found that the association between Beijing genotype and MDR became much weaker when it was adjusted for differences in resistance to streptomycin with or without resistance to isoniazid. This suggests that streptomycin resistance is “on the causal pathway”, i.e. that the association between Beijing genotype and MDR is mediated by, or conditional upon, resistance to streptomycin. In addition, when we stratified this association by presence or absence of streptomycin resistance it appeared to exist only among strains that were also resistant to streptomycin. Taken together these findings strongly suggest that in Vietnam streptomycin resistance is a prerequisite for Beijing genotype strains to become MDR. A role of streptomycin-resistance in the association between Beijing genotype and MDR has been suggested in several outbreaks of MDR-TB, including in New York City and Kenya (in which 100% and 71%, respectively, of the MDR-TB Beijing/W strains were also streptomycin-resistant) [42], [43].

These findings raise the hypothesis that the association of Beijing genotype with MDR is due to out-selection of streptomycin-resistant Beijing strains that do not display reduced reproductive fitness in settings with high drug pressure for streptomycin. This is the case in Vietnam, where streptomycin has been part of the standard treatment regimen for new and/or previously treated TB patients since 1986. It is likely also the case in many countries of the Former Soviet Union, where streptomycin is part of the standard first-line retreatment regimen and the proportion of TB patients who have been previously treated is high [1]. As we showed previously for Vietnam, co-existing isoniazid resistance then strongly increases the risk of treatment failure [44], and thereby for amplification to MDR. Whether this phenomenon reflects a single, clonally expanded Beijing strain or several strains requires further molecular studies, as do the specific mutations that confer streptomycin-resistance in these strains. In our analyses stratified by genotype we did not find any association between extent of clustering and rifampin resistance or MDR. It may be that Beijing strains that were streptomycin-resistant in addition to MDR have higher transmission fitness than strains that are MDR only, but numbers were too small to assess this.

Our study has a number of limitations in addition to those mentioned. We assumed for the non-EAI, non-Beijing (“other”) genotype strains for which we had no RFLP information that these were all of different RFLP types, while 16% were estimated to have similar RFLP patterns. This will have provided a minimum estimate of clustering of these strains only, and is a probable explanation why clustering among these strains was less than among EAI or Beijing strains. We excluded strains with VNTR types that had double alleles in ≥1 loci as mixed infections. However, some of these minor allele differences could represent evolution of the VNTR type within a single strain over time, and we may thereby have wrongfully excluded them. In addition, we did not collect data on HIV, although some studies suggested the HIV is a risk factor for clustering of TB patients [45], [46]. Nevertheless, since the estimated HIV prevalence among TB patients in our site was less than 1% this is unlikely to have affected our results [47].

## Conclusions

While this study was inconclusive with respect to overall differences in transmissibility between *M. tuberculosis* strains of the Beijing genotype and strains of the East-African Indian lineage. While we thus have no evidence that Beijing strains have a fitness advantage over other genotype strains, our data do indicate that Beijing strains retain their fitness if they are resistant to streptomycin, whereas East African Indian strains do not. Streptomycin-resistant Beijing strains even showed increased reproductive fitness when compared to streptomycin-susceptible Beijing strains. This suggests a selective advantage for these strains in treatment programs that include streptomycin in their standard regimens, which is further supported by out finding that streptomycin resistance is a prerequisite for an increased risk of MDR among Beijing strains in this setting. Tuberculosis control programs in settings where similar associations exist should reconsider the use of streptomycin.
